# Muscle Oxygen Changes following Sprint Interval Cycling Training in Elite Field Hockey Players

**DOI:** 10.1371/journal.pone.0120338

**Published:** 2015-03-25

**Authors:** Ben Jones, David K. Hamilton, Chris E. Cooper

**Affiliations:** 1 Centre for Sports and Exercise Science, School of Biological Sciences, University of Essex, Colchester, United Kingdom; 2 Director of Performance Science, USA Field Hockey, 2913 Spooky Nook Road, Manheim, Pennsylvania, United States of America; The University of Queensland, AUSTRALIA

## Abstract

This study examined the effects of Sprint Interval Cycling (SIT) on muscle oxygenation kinetics and performance during the 30-15 intermittent fitness test (_IFT_). Twenty-five women hockey players of Olympic standard were randomly selected into an experimental group (EXP) and a control group (CON). The EXP group performed six additional SIT sessions over six weeks in addition to their normal training program. To explore the potential training-induced change, EXP subjects additionally completed 5 x 30s maximal intensity cycle testing before and after training. During these tests near-infrared spectroscopy (NIRS) measured parameters; oxyhaemoglobin + oxymyoglobin (HbO2+ MbO2), tissue deoxyhaemoglobin + deoxymyoglobin (HHb+HMb), total tissue haemoglobin (tHb) and tissue oxygenation (TSI %) were taken. In the EXP group (5.34±0.14 to 5.50±0.14m.s^-1^) but not the CON group (pre = 5.37±0.27 to 5.39±0.30m.s^-1^) significant changes were seen in the 30-15_IFT_ performance. EXP group also displayed significant post-training increases during the sprint cycling: ΔTSI (−7.59±0.91 to −12.16±2.70%); ΔHHb+HMb (35.68±6.67 to 69.44±26.48μM.cm); and ΔHbO2+ MbO2 (−74.29±13.82 to −109.36±22.61μM.cm). No significant differences were seen in ΔtHb (−45.81±15.23 to −42.93±16.24). NIRS is able to detect positive peripheral muscle oxygenation changes when used during a SIT protocol which has been shown to be an effective training modality within elite athletes.

## Introduction

Adaptations in skeletal muscle function following specific training stimuli within elite athletes is an area of exercise physiology that is poorly understood. Near Infrared Spectroscopy (NIRS) is able to provide information about the changes in oxygenation and haemodynamics in muscle tissue based on the oxygen dependent characteristics of near infrared light [1]. Detailed reviews of NIRS methodology, medical applications and potential limitations have been published previously [2, 3]. Due, in part, to the development of portable wireless devices, the use of NIRS technology is gaining in popularity as an application to observe changes in muscle metabolism and muscle oxygenation during exercise [2, 3]. NIRS has been successfully and reliably used in both the laboratory [4] and in an applied sports setting [5], with specific measures such as muscle oxygenation level, deoxygenation rate and reoxygenation rate being utilised for the assessment of muscle oxidative function, following specific training interventions [6, 7]. NIRS studies have shown rapid skeletal muscle desaturation profiles during dynamic sprint exercise [8, 9]. Two NIRS signals have proved especially useful. The change in the concentration of deoxyhaemoglobin and deoxymyoglobin (HHb+HMb) signal has been suggested to be a measurement of changes in muscle oxygen extraction [10, 11]; whereas the measure of tissue oxygen saturation (TSI %) has been suggested to report upon the balance of oxygen delivery and oxygen consumption within the muscle [6, 12]. Alterations in either parameter following an exercise training intervention would potentially indicate a change in skeletal muscle oxidative capacity. Bailey and colleagues (2009) compared the effects of short term (two weeks) high intensity training (HIT) and endurance training (END) upon muscle oxygen kinetics in healthy males during step incremental moderate and severe intensity cycle exercise. HHb+HMb kinetics were found to be faster at the onset of exercise and the amplitude of the HHb+HMb response was increased in both exercise intensity domains following HIT training. The NIRS technique has also previously been utilised to detect improvements in muscle aerobic capacity following training with recent studies showing that both a two and a six week period of HIT (cycling & running) have resulted in improvements in aerobic power, Peak oxygen uptake (VO_2_) and maximal aerobic velocity together with increases in skeletal muscle deoxygenation capacity in untrained males [13, 14]. NIRS as a tool has shown promise as a reliable, non-invasive local measure of improved oxygen delivery and consumption in untrained populations following HIT training programs. Is it therefore postulated that NIRS will provide valuable information regarding individual elite athletes following specific training protocols.

Field hockey is a highly demanding intermittent effort sport requiring a myriad of high intensity actions. Global positioning Satellite (GPS) analysis has shown that outfield players can cover up to 6–12km, with up to 62 sprint efforts per game [15]. It is therefore appropriate to view field hockey as an aerobically demanding sport, with frequent although brief superimposed anaerobic efforts [16]. High intensity actions compose only a small percentage of match performances (5–8%) [17, 18], yet it is the successful completion of these high intensity actions that can potentially determine the outcome of most field hockey matches [19]. It is clear that hockey by nature requires a high level of aerobic fitness, a quality that when well-developed will facilitate an athlete’s ability to cover greater distances during a match and recover quicker between repeated high intensity efforts [20].

Sprint interval cycling (SIT) is a form of high intensity interval training [21] that is commonly used by elite athletes to enhance performance [22]. It is accepted that SIT elicits significant benefits to athletes through improvements in skeletal muscle together with positive adaptations in cardio-respiratory and metabolic function [23]. Adaptations within the muscle such as an increase in mitochondrial enzyme activity, increased capillarisation and mitochondrial biogenesis are thought to be responsible for an improved oxidative capacity within the muscle tissue [10].

The purpose of this study is; (i) to assess the individual effects of SIT on NIR muscle oxygen kinetics within a highly trained cohort of athletes; (ii) to assess whether one SIT session per week over 6 weeks is sufficient to improve performance during the 30-15 intermittent fitness test (30-15_IFT_) compared to the control group.

Previous exercise test assessments of SIT have focused solely upon aerobic endurance capacity (VO_2_max, total work volume) [22, 24]. However, during intermittent effort sports, such as hockey the energy demands are more varied and require the utilization of all the energy pathways recurrently, with a growing dependence upon aerobic metabolism as a match progresses [16]. Energy expenditure during maximal intensity exercise tests has contributions from both anaerobic and aerobic metabolism [25]; and repeated all-out thirty second cycle efforts interspersed with four minutes of recovery, rely progressively upon aerobic energy provision [23]. It was therefore felt that a repeated maximal intensity exercise test would be an appropriate laboratory procedure to test enhancements in aerobic energy provision targeted towards this specific type of sport.

We therefore hypothesized that following a SIT training protocol optical measurements would indicate a greater muscle deoxygenation as the result of an enhanced muscle oxygen extraction capacity. This response would be detectable by NIRS, in elite athletes, during maximal intensity exercise. This would also be associated with an improved exercise performance during an intermittent sprint fitness test.

## Methods

Twenty five elite women hockey players of Olympic standard were randomly selected into an experimental (EXP, n = 10) training group (mean ± SD: age 20.6 ± 0.9 yrs; height 187 ± 0.6 cm; weight 65.0 ± 4.3 kg; quadriceps skin fold 9.2 ± 1.5 mm) and a control (CON, n = 15) group (mean ± SD: age 21.4 ± 1.0 yrs; height 185 ± 4.1 cm; weight 67.3 ± 2.1 kg; quadriceps skin fold 9.6 ± 0.8 mm) for assessment. The data for four subjects in the EXP group was subsequently excluded due to injury and illness. Thickness of the adipose layer of tissue overlying the quadriceps muscle was measured using a skinfold caliper (British Indicators, Harpenden, UK) and no significant differences between baseline and post-training descriptive measures were found (P = 0.33).

### Ethics Statement

All subjects gave their written informed consent before taking part in the study which was approved by the ethics committee of the University of Essex.

### Experimental Procedures

All testing was performed in a controlled indoor environment at the England Institute of Sport (EIS) strength and conditioning training facility. Experimental group subjects were asked to complete a repeat sprint cycle test upon a Wattbike Pro (450±106 W) at air brake resistance level 3, magnet setting 1 with haemoglobin variables of the *vastus lateralis* muscle (near infrared spectroscopy) being recorded throughout the testing period. Prior to testing, the zero was calibrated for each Wattbike ergometer in accordance with the manufacturer’s recommendations. Saddle and handlebar height and position were recorded and replicated for each trial; subjects used the same Wattbike ergometer for pre and post testing. The reliability of power measurements obtained for trained athletes using a Wattbike Pro during high intensity exercise, has shown to be highly reproducible (CV = 2.6%, 95% CI = 1.8–5.1%) [26]. During each session the rating of perceived exertion (RPE) was recorded for each subject during each sprint. Subjects were instructed to cycle in an upright position (upright torso) with the gluteal muscles maintaining contact with the seat at all times. The warm up protocol consisted of 3 x 55 seconds (s) of steady state cycling interspersed with 3 x 5 s sprints efforts. The repeated maximal intensity cycle test procedure consisted of 5 x 30 s maximal sprint efforts interspersed with 4 minutes of recovery. Individual measurements of fatigue index (FI%), average and peak power (w) were calculated for both pre and post cycle testing. FI was calculated as;

$$\text{FI}=\text{100}*\left({\text{P}}_{\text{best}}-{\text{P}}_{\text{worst}}\right)/{\text{P}}_{\text{best}}$$

where (P) refers to the power score. The FI indicates a drop off in performance from the best to the worst power performance [27] during the maximal intensity cycling protocol. Peak and average power were calculated as the mean of the five sprints.

On a separate testing day all subjects (EXP vs. CON) were also required to perform a 30-15 intermittent fitness test (30-15_IFT_) before and after the training intervention. The test consisted of 30 seconds of shuttle runs with 15 seconds of passive recovery [28]. The test starting velocity was set at 8 km.h^−1^, and the speed was increased by 0.5 km.h^−1^ at 30 second intervals. The test required subjects to run back and forth between two lines (40 m apart) at a pace directed by a pre-recorded audible signal. Subjects were instructed to complete as many stages as possible, with test cessation occurring when a subject could no longer maintain the required running speed. The final running rate velocity (_VIFT_) achieved during the 30-15_IFT_ test has been shown to provide a high within-day reliability as assessed by intraclass correlation coefficient (ICC = 0.96, Typical error = 0.33, 95% CI = 0.26–0.56 km.h^−1^) [29]. NIRS analysis of the effect of training on muscle oxygen extraction and utilisation was carried out during maximal repeated sprint cycle exercises.

### Training Intervention

The exercise training intervention for the EXP group consisted of six sessions of repeated sprint interval sessions over a six week period (one session per week) in addition to the subject’s normal training week (5 x field hockey practice and 3 x weight sessions per week). The sprint interval training (SIT) was performed as previously described [13, 30] and consisted of repeated 60 (s) high intensity sprint efforts upon a Wattbike Pro at a workload that corresponded to the peak power achieved during the pre-test maximal intensity cycle testing (450±106 W). This was interspersed with 75 (s) of active recovery low cadence cycling (45±10 W) between efforts. Subjects completed eight high-intensity intervals during the first two training sessions, 10 intervals during the third and fourth sessions, and 12 intervals on the final two sessions. A 3 min warm-up at 30–50 W interspersed with a 5 (s) sprint at the end of each minute was performed prior to testing and the total time commitment during each training session therefore ranged from∼20 to 30 minutes. This time frame included the warm-up, the training intervention and recovery, which resulted in a total commitment of less than 3 h of exercise over 6 weeks. Each session was led by the team strength and conditioning coach and subjects were given strong verbal encouragement to maintain workload efforts throughout. Rating of perceived exertion (RPE) and power output data was taken for each training session to confirm training intensity.

### NIRS Measurements

During sprint cycling, muscle oxygenation of the *vastus lateralis* muscle of each subject’s dominant leg was continuously monitored using a commercially available portable NIRS apparatus (PortaMon, Artinis, Medical Systems). This device is a 2-wavelength, continuous wave system, which simultaneously uses the modified Beer-Lambert law and spatially resolved spectroscopy (SRS) methods. Using such a 2 wavelength system means it is not possible to separate the changes in light absorbed by haemoglobin or muscle. The total muscle myoglobin content does not change in the course of an acute study, therefore it was possible to use the difference in absorption characteristics of light at 750 and 850nm to report changes in tissue oxyhaemoglobin + oxymyoglobin (HbO_2_+ MbO_2_), tissue deoxyhaemoglobin + deoxymyoglobin (HHb+HMb), total tissue haemoglobin (tHb) and tissue oxygenation (TSI %).

Values for (HbO_2_+ MbO_2_), (HHb+HMb) and tHb are reported as a change from baseline (30 s averaging before each test) in micromolar-centimeter units (μM.cm). Additionally, the tissue oxygen saturation index (%TSI) was calculated using spatially resolved spectroscopy (SRS) methods. TSI reflects the dynamic balance between O_2_ supply and consumption. The portable device was positioned on the belly of the *vastus lateralis*, midway between the greater trochanter and the lateral epicondyle of the femur. To ensure the optodes and detector did not move relative to the subject’s skin, the device was fixed into position using a waterproof adhesive tape which was then secured with a black neoprene sports strapping. A surgical marker pen was used to mark probe placement in order to identify any device movement during testing. No sliding was observed at the end of any measurement in any subject. To ensure accurate repositioning for subsequent testing the same International Society for the Advancement of Kinanthropometry (ISAK) qualified researcher performed anthropometric measurements for device placement. Care was taken to ensure that this method of fixation was sufficient to prevent movement of the device during testing without limiting the subject’s movement in any way. During all tests, the NIRS system was connected to a personal computer via bluetooth for data acquisition (10Hz), analogue-to-digital conversion and subsequent analysis.

### NIRS data and assessment

To fully observe a complete haemodynamic profile from the NIRS data, it was important to report on all NIRS measurements. However for this study we focused predominantly on the changes in the %TSI and the deoxyhaemoglobin and deoxymyoglobin (haemoglobin + myoglobin) signals to allow comparisons with previous results [6, 8]. Both TSI and (HHb+HMb) measurements of oxygenation have been considered to be representative as surrogate measures of oxygen extraction [31]. Additionally, both have been regarded as essentially blood volume insensitive during exercise [10] and thus cannot be misinterpreted by blood volume changes that can occur during sprint cycling. Maximum and minimum values were calculated as a three second average surrounding the highest and lowest values during each of the five sprint periods. The Δ in TSI, (HHb+HMb), (HbO_2_+MbO_2_) and tHb was therefore calculated as the maximum value minus the minimum value during each sprint e.g. TSI Max—TSI Min = ΔTSI. Additionally time to peak desaturation was calculated as the time in seconds (s) at which the minimum TSI value was obtained during each sprint. Reliability of tissue oxygenation index and deoxyhaemoglobin responses obtained by NIRS has previously been evaluated via coefficient of variance (CV) and intraclass correlation coefficient (ICC). The repeated measurements for the same subject, inter-day, was found to be within acceptable limits (CV = ∼7–11%) [4] (ICC = 0.99 CI = .98–.99) [32] for skeletal muscle [33].

### Statistical analysis

Descriptive statistics are presented as a mean ± SD unless otherwise stated. Each variable was examined with Kolmogorov-Smirnov normality test. For pre- versus post-training comparisons, all NIRS variables, power indices and 30-15_IFT_ scores were analyzed using either paired (2-tailed) *t*-tests or Wilcoxon signed rank test when the sample normality test failed as per Bravo and colleagues [34]. The level of significance for analyses was set at p < 0.05. The standardized difference or effects size (ES) in each performance measure was calculated using the pooled standard deviation. Data was also assessed for clinical significance using an approach based on the magnitudes of change with 90% confidence limits (90%CL) [35]. Pearson’s product–moment correlation analysis was used to compare association between optical measures and performance. All analyses were performed using SPSS 19.0 software for windows (SPSS, Inc., Chicago, IL, USA).

## Results

**Fig 1:**
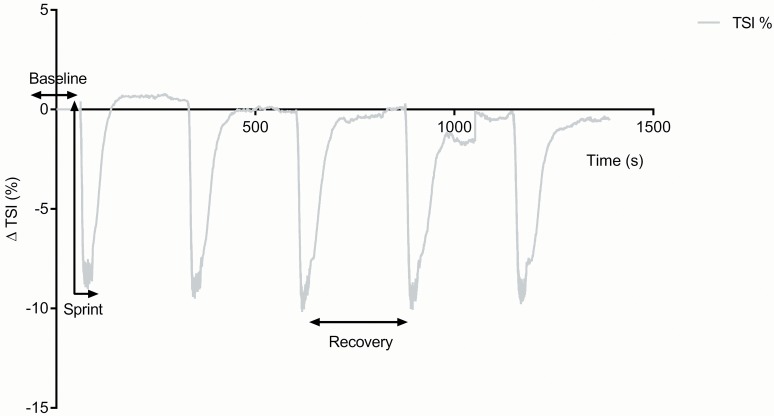
Pre-training mean tissue saturation index trace (ΔTSI %) during the test protocol.

**Fig 2:**
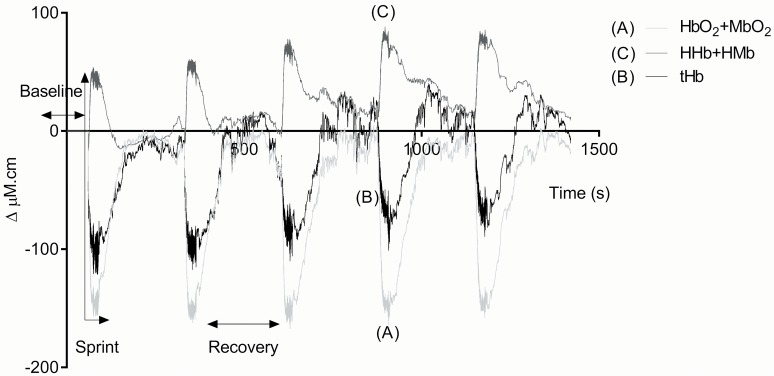
Group mean pre-training data trace for ΔHHb+HMb, ΔHbO_2_+MbO_2_, & ΔtHb.

**Fig 3:**
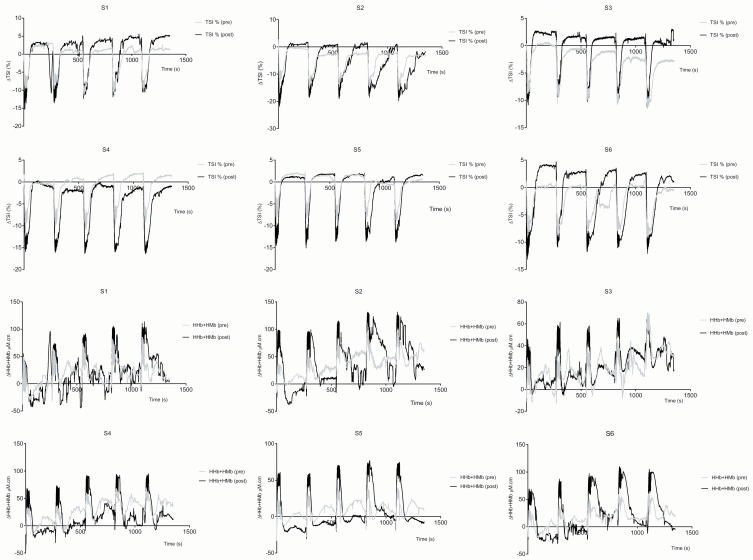
Experimental group individual pre vs. post training ΔTSI and ΔHHb+Mb values.

Fig. 1 & Fig. 2 illustrate the NIRS detected changes (group averaged data) for the pre-training test (the general trend was similar for both tests). A decrease in TSI was seen immediately upon the start of the maximal intensity cycling test. This recovered rapidly following the end of the test, in some cases overshooting the baseline (a hyperaemic effect). This drop in TSI was caused by a fall in HbO_2_ + MbO_2_ and a rise in HHb+HMb. The total volume of blood in the muscle (tHb) also fell immediately upon exercise start. Table 1 summarizes the effect of training on all the muscle optical parameters. The tissue became significantly more deoxygenated post training, with significant changes in both HbO_2_+MbO_2_ and HHb+HMb. However, no significant change was seen in muscle blood volume (tHb). Fig. 3 shows the experimental group individual data for the changes in TSI and HHb+HMb amplitude for pre vs. post repeated maximal intensity cycle tests.

**Table 1:** Individual averaged data (sprint 1-sprint 5) values for pre vs. post training; delta Tissue Saturation Index (ΔTSI (%)) and delta deoxyhaemoglobin + deoxymyoglobin (ΔHHb+HMb), delta oxyhaemoglobin + oxymyoglobin (ΔHbO_2_+MbO_2_) and delta total haemoglobin (ΔtHb) in micromolar-centimeters (μM·cm).

	PRE	POST	PRE	POST	PRE	POST	PRE	POST
**Player**	ΔTSI (%)	ΔTSI (%)	ΔHHb+HMb	ΔHHb+HMb	ΔHbO_2_+MbO_2_	ΔHbO_2_+MbO_2_	ΔtHb	ΔtHb
**S1**	−7.77	−9.64	44.32	60.80	−77.12	−98.56	−38.72	−42.24
**S2**	−9.19	−16.92	41.92	111.84	−80.0	−144.48	−45.92	−39.36
**S3**	−7.30	−9.65	28.0	31.04	−84.96	−90.56	−64.16	−62.40
**S4**	−7.36	−13.14	31.04	67.04	−74.72	−123.84	−49.44	−60.32
**S5**	−6.40	−11.90	30.88	64.96	−47.04	−84.48	−20.32	−19.84
**S6**	−7.49	−11.71	37.92	80.96	−81.92	−114.24	−56.32	−33.44
**Mean± SD**	−7.59 ±0.91	−12.16* ±2.70	35.68 ±6.67	69.44* ±26.48	−74.29 ±13.82	−109.36* ±22.61	−45.81 ±15.23	−42.93 ±16.24

*Denotes significance. Significant post-training increases (p < 0.05) were seen in ΔTSI; P = .003, ES = 0.75 ± 0.28; ΔHHb+HMb (p < 0.05); P = .016, ES = 0.65 ± 0.32; ΔHbO_2_+MbO_2_; P = .028, ES = 0.68 ± 0.45. No significant differences were seen in ΔtHb; P = .563, ES = 0.09 ± 0.29.

**Fig 4:**
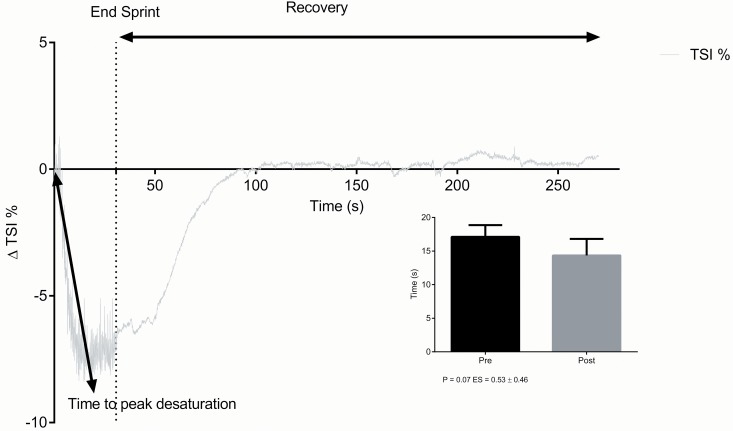
A non-significant decrease in time to peak desaturation was found post training; P = 0.07, ES = 0.53 ± 0.46.

Fig. 4 shows time to peak desaturation calculation and group average pre vs. post training time to peak desaturation time (s).

**Fig 5:**
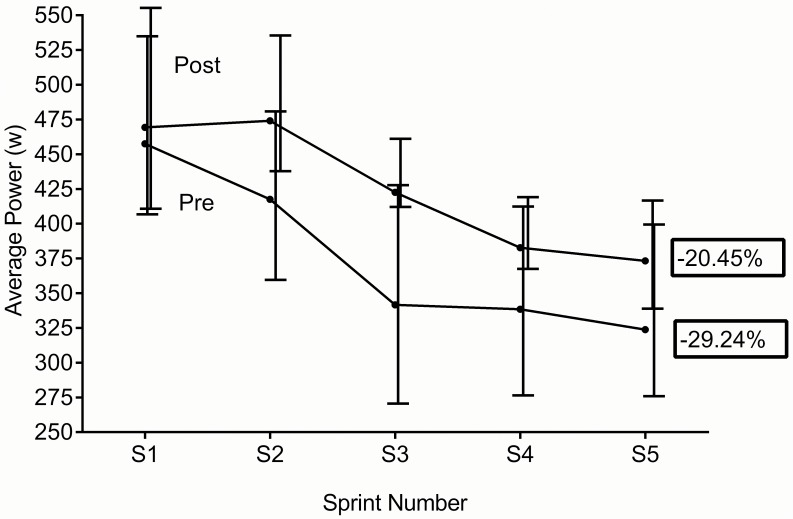
Group mean data (sprint 1-sprint5) pre vs. post average power & fatigue index (90%CL).

**Fig 6:**
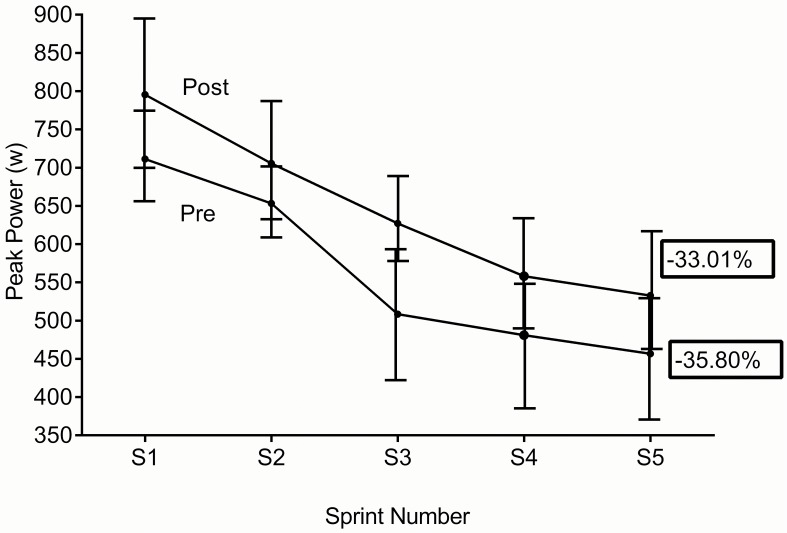
Group mean data (sprint 1- sprint5) pre vs. post peak power and fatigue index (90%CL), non-significant reductions in fatigue index were seen.

Table 2 displays individual values for pre vs. post training. Non-significant changes were seen for average and peak power. Fig. 5 (average power) and Fig. 6 (peak power) shows the grouped data for each sprint. In both cases there was no significant reduction in fatigue index post training. No significant difference was seen in subjects rating of perceived exertion (RPE) between tests.

**Table 2:** Individual average and peak power values for pre vs. post training.

	Average Power (w)	Average Power (w)	Peak Power (w)	Peak Power (w)	RPE Borg (1–10)	RPE Borg (1–10)
**Player**	PRE	POST	PRE	POST	PRE	POST
**S1**	278.6	460.8	485.0	776.0	7.6	9.6
**S2**	289.2	418.8	474.0	647.2	8.8	7.6
**S3**	406.8	391.4	551.0	521.4	7.8	8.2
**S4**	491.8	494.2	674.6	657.6	7.6	8.8
**S5**	411.6	398.6	624.8	645.6	7.8	8.6
**S6**	377.2	383.0	563.8	615.4	7.0	9.2
**Mean±SD**	**375.87±73.77**	**424.47±40.20**	**562.20±77.99**	**643.80±81.80**	**7.76±0.58**	**8.66±0.71**

Non-significant increases were seen in average, peak power output and subject rating of perceived exertion; P = .221, ES = 0.37 ± 0.53. P = .172, ES = 0.45 ± 0.57. P = .135, ES = 0.57 ± 0.64.

**Fig 7:**
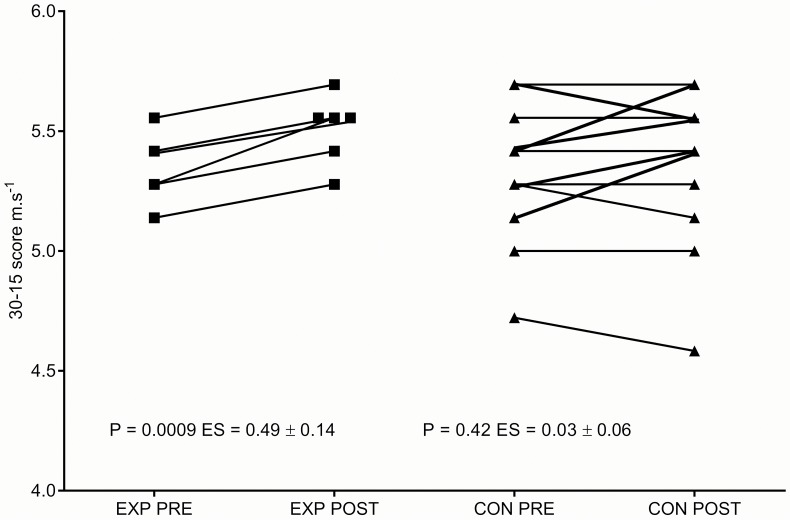
Individual pre vs. post Experimental and Control group 30-15_IFT_ final running rate velocity scores.

Changes in the 30-15 Intermittent Fitness Test (30-15_IFT_) revealed significant EXP group increases in final running rate velocity (_VIFT_) (m.s^−1^); pre 5.34 ± 0.14 vs. post 5.50 ± 0.14 m.s^−1^; P = 0.0009, ES = 0.49 ± 0.14 (90% CL), compared with non-significant changes in the CON group; pre 5.37 ±0.27 vs. post 5.39 ± 0.30 m.s^−1^; P = 0.42, ES = 0.03 ± 0.069. Fig. 7 displays that all EXP group subjects increased their final running rate velocity with larger variability seen within the CON group. No significant correlations were observed between optical and 30-15_IFT_ performance measures; Δ% HHb+HMb vs. Δ%30-15_VIFT_, R^2^ = 0.19; Δ% TSI vs. Δ%30-15_VIFT_, R^2^ = 0.38. Significant correlations were found between time to peak desaturation vs. peak power output; P = 0.03, R^2^ = 0.7 ± 0.44 and Δ% peak power vs. Δ% 30-15_VIFT_; P = 0.03, R^2^ = 0.7 ± 0.52.

## Discussion

The primary findings of this study reveal that, in agreement with our hypothesis, significant changes were seen in optical measures following SIT training, indicative of an increased muscle oxygen extraction capacity. This is represented by both the TSI (%) and HHb+HMb signals. The significant changes in the group data is reflected in every individual with each athlete exhibiting an increased desaturation of TSI (%) and a rise in HHb+HMb during post-training sprint efforts. Additionally a significant increase in final running rate velocity (_VIFT_) during the 30-15_IFT_ was seen in the EXP group but not within the CON group. The fact that, six sessions (one per week) of SIT is able to improve 30-15_VIFT_ performance within an already highly trained group of athletes, compared to their training counterparts, is potentially very valuable. Furthermore, it appears this off-feet modality is able to provide sufficient stimulus to promote aerobic adaptation, whilst minimising the risk of injury due to excessive training load. This is of great importance within elite training groups.

To our knowledge this is the first study which shows consistent changes in NIRS parameters and individual performance in all elite athletes following a training program such as SIT. This demonstrates the potential of utilizing NIRS as a tool to monitor local muscle oxygen flow and metabolism and also the value of SIT as a training modality in elite athletes.

### NIRS Assessment

Concomitant with training induced performance improvements significant alterations in typical markers of muscle oxidative potential such as cytochrome *c* oxidase and citrate synthase [24] have been noted in previous studies using muscle biopsy samples [22, 36]. In contrast to these invasive markers, in this study we tested whether a non-invasive measure of blood flow and oxygen metabolism (NIRS) could have the potential for real-time and continuous feedback on the training process.

What aspects of the mechanism of SIT-induced improvements in muscle oxygen delivery and metabolism are accessible by NIRS measurements? Following training, significant increases were seen in TSI (%) and HHb+HMb, with a significant decrease observed in HbO_2_+MbO_2_ (group & individual data). tHb however remained unchanged. Both trend (HHb+HMb, HbO_2_+MbO_2_) and spatially resolved (TSI %) optical data show deoxygenation changes independent of blood volume, consistent with a post training increase in muscle oxygen consumption and oxygen extraction during the maximal intensity cycling exercise test. The fact that each individual subject demonstrated similar NIRS signal patterns suggests that these parameters could be useful reporters of training adaptations. Based on the assumption that an increase in the desaturation of muscle oxygen saturation (TSI) and an increment in the HHb+HMb amplitude overall represents an increase in muscle oxygen extraction. These observed changes can be termed as ‘positive peripheral muscle oxygen adaptations’ which have specifically occurred in response to training. This finding is in agreement with previous studies [13, 14]. The physiological mechanisms responsible for this enhanced extraction capacity are likely multifactorial and complex. Due to the non-invasive approach to this study, a lack of histological evidence limits mechanistic cause to speculative assessment. Current literature regularly states an increased mitochondrial biogenesis as the suggested adaptation to explain the increased ability to extract oxygen following both SIT and endurance (END) training [31]. Mitochondrial biogenesis is reported to occur through repeated contractile activity in actively recruited muscle fibers [37]. The higher exercise intensity provided during SIT will increase the likelihood of favorable adaptations occurring in both type one and type two fibers [38] as opposed to the generally lower intensity of traditional END training. This is one of the favorable aspects of SIT that is important for intermittent high intensity effort sports that requires athletes to possess high aerobic capacities, coupled with the ability to greatly tax their anaerobic capability [16]. It has also been suggested that the increased level of muscle deoxygenation and near maximal stress to the energy pathways that occur during SIT exercise could act as a potential initiator mechanism for mitochondrial biogenesis [39]. The profile of muscle deoxygenation that occurred during the repeated maximal intensity exercise test, matched muscle desaturation patterns that have been previously described during comparable repeat sprint tests [8]. A nadir was generally achieved around 10–15 seconds, suggestive of maximal desaturation with a plateau occurring thereafter. As the SIT protocol consisted of sprint efforts of 60 seconds in duration, prolonged and maximal desaturation periods are likely to have occurred during training sessions.

### NIRS Reproducibility

Identifying the characteristic measurement error associated with ΔTSI (%) and ΔHHb+HMb will have important implications for this and future studies in which any training intervention is utilized. This is especially important as often only small performance changes (<1%) in response to training are commonly seen within elite athlete [40]. Device measurement variance-to-training adaptation (signal-to-noise) overlap can make the interpretation of the magnitude and benefit of imposed interventions difficult [41]. This becomes paramount if a NIR measurement is to be used as an individual training monitoring tool, as of yet this application of portable NIR technology is yet to be established. To date, little attention has focused upon the reproducibility of the raw NIRS parameters (ΔHbO_2_+MbO_2_, ΔHHb+HMb and ΔtHb) [42] and there is an absence of normative data within the literature. Reproducibility measurements have primarily focused upon NIR calculated metrics such as muscle oxygen consumption (mVo_2_) and recovery based measurements. For example, previous research groups have used repeated arterial occlusion techniques to provide measurements of mitochondrial oxygen consumption [43, 44]. Unfortunately the practicality of these measures during dynamic exercise is restricted with varying estimates of reproducibility shown (Coefficient of Variance (CV) ∼6–20% [45], (CV∼15–25%) [46]. In the present study it is suggested that an interpretation of the raw data signal, including the totality of NIRS measurements enables sensible inferences to be made of training effects on individual athlete’s muscle oxygen extraction and consumption.

Tissue oxygen saturation (StO_2_, TOI) measurement error has been reported by Muthalib et al (2010) and Austin et al (2005) during repeated maximal intensity isometric contractions and cycle exercise with a CV of ∼7–11% and single trial coefficients (R = .99 CI = .98–.99, RMSE = 1.6). Parameter estimates for the accuracy for the HHb+HMb signals has been carried out by Spencer at al. (2011). Repeated measures ANOVA analysis revealed no significant difference in ΔHHb+Mb amplitude following multiple inter and intra-day moderate intensity exercise transitions. Unfortunately the authors did not include ΔHHb+HMb amplitude metric within their further analysis. However the raw data presented in arbitrary units (a.u.) shows averaged amplitude ΔHHb+HMb scores of; 7.5 ± 6.9, 8.0 ± 7.1 and 7.0 ± 6.1 following repeated exercise tasks, potentially indicating a high reproducibility of measurement. Based upon current literature we are therefore unable to provide a quantitative measure of the confidence limits, of the associated error measurement related with the NIRS parameters presented within this study. However, based upon the above estimates, we are confident that the observed changes in NIR parameters within this study are the result of training adaptation and not measurement error.

### Individual Assessment

Can NIRS be used for individual intervention response profiling? To the best of our knowledge only Neary et al [47] has previously published individual NIR trace data (deoxyhaemoglobin + myglobin (Hb/Mb-0_2_)) following a training intervention. Neary and colleagues displayed individual Hb/Mb-0_2_ traces for eight male cyclists following a three week HIT endurance program. Group averaged data during the post-training 20km time trial showed significantly greater Hb/Mb-0_2_ amplitude. Individual responses revealed that five cyclists exhibited significantly greater deoxygenation, one cyclist had a reduced deoxyhaemoglobin profile with two subjects showing no change. This study highlights the importance of individual analysis and supports the premise that trend data cannot be utilised for individual exercise prescription at an elite level. In light of this, individual analysis was carried out within the current data set, however, no clear relationships between optical and performance data were identified. Certainly individuals who displayed the greatest ‘positive’ changes in NIR indices did not demonstrate as pronounced changes in performance parameters. For example; Subject 1 displayed the greatest improvements in 30-15_VIFT_, peak power, average power and fatigue index, but showed the 2^nd^ smallest change in ΔTSI (%) and ΔHHb+HMb. Subject 5 reported the greatest change in ΔTSI (%) and ΔHHb+HMb, but displayed the second smallest change in power and the group average improvement score in 30-15_VIFT_. Admittedly, within such a small cohort any correlations may be difficult to identify and any interpretation must be viewed with appropriate caution. Buchheit & Ufland (2011) reported improvements in muscle reoxygenation rate, performance decrement and maximal aerobic running speed following eight weeks of endurance training in moderately trained men. No such relationships were found between NIR and running performance measures within the current study. Inherent differences between the current study and Buchheit and Ufland related to training load and modality are potential explanations for the seen differences. It is possible that the greater metabolic cost of run exercise compared to cycling facilitates a greater physiologic change that can be identified by NIRS monitoring. A strong correlation (R^2^ = 0.7) was observed between time to peak desaturation and peak power output during the repeated maximal intensity cycle test. Additionally, a significant correlation (R^2^ = 0.7) was found between Δ% peak power and Δ% 30-15_VIFT_. The SIT protocol was designed in order to expect an increase in muscle oxidative capacity rather than an improvement in power indices. This is perhaps evidenced by the mixed individual power output responses, but more uniformed individual response in NIR metrics during the repeated cycle test. The correlation between %Δ peak power and %Δ 30-15_VIFT_ is perhaps not unsurprising as it is currently debated to what extent the availability of oxygen at the local (muscle) level has on a subjects ability to perform repeat sprint activity[48]. The major component of peak speed is suggested to be largely determined by neuromuscular factors (motor unit recruitment, synchronicity) [27]. However the ability to resist fatigue (% decrement), replenish energy substrates (ATP, PCr), and the efficiency of the removal of inhibitory metabolites (H^+^, P_i_) (all of which are oxygen dependant processes) must also contribute to sprint repeatability [49]. It is a combination of all of these factors that have led to an increased interest in the ability of SIT to increase aerobic capacity and skeletal muscle oxidative capacity.

We conclude that concurrent observations of improved exercise performance and significant changes in optical data within the current study support the ability of NIRS to explore and report on peripheral muscle adaptations in response to training stimuli in elite athletes. This study shows that further research in this still poorly understood area is required if NIRS measurements are able to inform and benefit training prescription.

### SIT Assessment

The SIT sessions utilised during the 6 week block appear to have provided sufficient training intensity and duration to elicit positive performance improvements (as evidenced by the 30-15_VIFT_ scores). The intensities selected within the training intervention were purposefully progressive to allow a constant training stimulus. Previous studies incorporating a similar SIT protocol have shown improvements in mean power, peak power and time trial performance in healthy [30] and untrained individuals [13]. It has been noted that both central and peripheral adaptations are required within the aerobic system in order to fully optimize an individual’s aerobic fitness. It is suggested that any training above 80% VO_2_max is sufficient to promote peripheral adaptations and changes within the muscle, namely; muscle capillarisation, mitochondrial proliferation and improved oxidative enzyme activity [20]. A recent study by Buchheit et al (2012) examining the physiological responses to a single SIT session (6 x 30sec maximal efforts, 2 minutes passive rest) found that subjects were able to reach values above 90% of peak VO_2_ and heart rate. However, based on the absence of experimental data on both central and peripheral factors, it is too speculative at present to provide a definitive mechanistic explanation for our observed improvements in performance.

### Limitations

Some inherent limitations to the NIRS technique and its technology have been reported [50]. The design of the current study was able to overcome the more common issues relating to adipose tissue thickness and movement artifact. A large percentage of adipose tissue over the site of interrogation can greatly influence light pathlength and make it difficult to quantify tissue oxygen [51]. Within this study subjects had what was considered to be a minimal layer of adipose tissue (9.2 ± 1.5 mm) making it unlikely that this would be a confounding issue, especially as no significant changes were seen in adipose layer thickness post training. Motion artifact can sometimes produce large ‘spikes’ in the oxygen measurements, usually due to an unsecure device attachment or the forceful muscle contractions [52] experienced during dynamic exercise. Cycle exercise provides a non-impact testing environment, which when coupled with secure device placement upon a large flat muscle area greatly reduces the appearance of motion artifact.

Due to the access constraints within elite sports, the present study is also limited by the low sample size (n = 6) of the EXP group, only the EXP group performing the repeated maximal intensity cycle testing and the lack of optical measurements taken during the 30-15_IFT_. There is a need to further validate the efficacy and appropriateness of a NIR measurement within a sporting environment and it is hoped that this preliminary study will encourage more frequent use of NIRS within elite sport, providing further insight into the physiological processes behind individual adaptations.

### Perspectives

Near Infrared Spectroscopy (NIRS) can measure non-invasively changes in muscle oxy and deoxyhaemoglobin concentrations. Previous studies in untrained [13] and trained [6] subjects have shown that NIRS has the ability to detect local muscle oxygenation changes in response to training. However, these studies are rarely performed on elite athletes and report primarily on group mean, not individual data. Several subjects are apparent non-responders, but it is not clear whether this is due to a genuine physiological variability in the response to training, inappropriate protocols to assess local muscle changes, or indeed errors in the NIRS measurement itself. These issues need to be addressed prior to NIRS being used to monitor the training progress of individual athletes, especially in those in the elite category where training responses are commonly less pronounced. We therefore performed a training study on the UK elite women’s field hockey team. We first demonstrated that our sprint interval training (SIT) program did indeed enhance performance. Having demonstrated this we measured the effect of SIT on exercise-induced NIRS-measured muscle oxygenation changes. Using a robust maximal intensity cycle test, significant training–induced changes were seen, consistent with enhanced muscle oxygen extraction. Crucially these changes were seen in all individuals. This study therefore opens up the possibility of using NIRS to track local, as opposed to global, changes in oxygen metabolism in individual subjects at all levels of sporting proficiency.
